# Maternal Immune Response During Pregnancy and Vertical Transmission in Human Toxoplasmosis

**DOI:** 10.3389/fimmu.2019.00285

**Published:** 2019-02-21

**Authors:** Fernando Gómez-Chávez, Irma Cañedo-Solares, Luz Belinda Ortiz-Alegría, Yevel Flores-García, Héctor Luna-Pastén, Ricardo Figueroa-Damián, Juan Carlos Mora-González, Dolores Correa

**Affiliations:** ^1^Laboratorio de Inmunología Experimental, Instituto Nacional de Pediatría, Secretaría de Salud, Mexico City, Mexico; ^2^Cátedras CONACyT-Instituto Nacional de Pediatría, Mexico City, Mexico; ^3^Servicio de infectología e Inmunología, Instituto Nacional de Perinatología, Mexico City, Mexico; ^4^Centro de Salud Gustavo A. Rovirosa, Secretaría de Salud, Mexico City, Mexico

**Keywords:** *Toxoplasma gondii*, human congenital toxoplasmosis, vertical transmission, cellular response, IFN-γ, TGF-β1, IgG subclasses, IgA

## Abstract

Toxoplasmosis is a parasitic zoonosis distributed worldwide, caused by the ingestion of contaminated water/food with the parasite *Toxoplasma gondii*. If a pregnant woman is infected with this parasite, it may be transmitted to the fetus and produce ocular, neurological, or systemic damage with variable severity. The strength and profile of mother's immune response have been suggested as important factors involved in vertical transmission rate and severity of clinical outcome in the congenitally infected fetus. The aim of this work was to evaluate a possible relation between the mother's immune response during pregnancy and congenital transmission to the fetus. We obtained peripheral blood from *T. gondii* infected pregnant woman and tested it for anti *T. gondii* (IgG1, IgG2, IgG3, IgG4, and IgA) in serum. Peripheral blood mononuclear cells (PBMCs) were isolated to analyze the *in vitro* effect of soluble *T. gondii* antigens on proliferation and production of cytokines. We found that IgG2-4 and IgA antibodies and lymphocytes proliferation, especially CD4^+^, CD8^+^, and CD19^+^ were positive in a higher proportion of cases in transmitter than in non-transmitter women. Furthermore, IgG2-3 and IgA anti-*Toxoplasma* antibody levels were higher in those mothers who transmitted the infection than in those who did not. Interestingly, a higher proportion of positive cases to IFN-γ and negatives to the immunoregulatory cytokine TGF-β, were related to *T. gondii* vertical transmission. Our descriptive results are consistent with the paradoxical previous observations in murine models of congenital toxoplasmosis, which suggest that an increased immune response that protects the mothers from a disseminated or severe disease, and should protect the fetus from infection, is positively related to parasite transmission.

## Introduction

*Toxoplasma gondii* is a foodborne pathogen which infects approximately one-third of all humans (1). Most individuals with toxoplasmosis show no clinical signs, however immunodeficient and congenitally infected patients may develop pathological conditions (2).

Congenital infection occurs due to vertical transmission of *T. gondii* during pregnancy, and although it is usually asymptomatic and self-limited in the mother, if the fetus is infected, he/she may develop variable clinical features, such as spontaneous abortion, stillbirth, hydrocephalus, macro or microcephalus, cerebral calcifications, retinochoroiditis, and other ocular or central nervous system alterations, which can manifest even years later in life (3).

It is widely known that in immunocompetent individuals, such as pregnant women, a Th1-type immune response represents the main effective response against the parasite (4, 5). Although there are some reports about *T. gondii* infected mother's immunity during pregnancy, it's role in promoting or inhibiting congenital transmission has not been directly tested (6–10).

Other important players involved in the control of *T. gondii* during acquired infections, are human IgG subclasses -predominantly IgG1- and their Fc receptors; importantly, specific IgG1 in infected mothers has been related to clinical problems in their congenitally infected babies; however, they were measured at delivery, when transmission already occurred (11).

Due to the lack of information about the specific lymphocyte populations, cytokines, and antibody subtypes induced by *T. gondii* in infected women during pregnancy and their relationship to vertical transmission, we performed the present study.

## Materials and Methods

### Ethical Aspects

This work was carried out in accordance with the World Medical Association's Declaration of Helsinki. It contains partial results from the project 060/2011, approved by the Research and Research Ethics Boards of the Instituto Nacional de Pediatría (INP), Mexico City, Mexico; registered at the Office for Human Research Protection of the NIH (http://ohrp.cit.nih.gov/search/search.aspx) with numbers IRB00008064 and IRB00008065. It was also approved by the INP Committee of Laboratory Animal Use and Care; approval is available upon request. The Instituto Nacional de Perinatología (INPer) also approved the project (number 212250–02231). All participants signed an informed consent, which explicitly stated that it was of low risk, considering that clinical management was not modified for the protocol. All newborns were clinically managed at INP according to national and international standards. Biosafety measures were carefully followed, in order to avoid technician's contamination with the *T. gondii* strain used to prepare the antigen, by using a level II biohazard hood (Labconco Purifier Class II Biosafety Cabinet, Labconco Corp., Kansas, MO) when working with the parasites. A well-controlled animal house is present at INP, where the mice are inoculated. In addition, to avoid, potentially contagious diseases (present in the women recruited), only trained personnel who wore gloves and face masks was authorized to take samples from patients.

### Parasite Antigen

*Toxoplasma gondii* tachyzoites (RH strain) were maintained in BALB/c mice by intraperitoneal passages. Peritoneal exudates from 40 mice were harvested and washed twice (720 × g, 10 min, 4°C) in PBS supplemented with a protease cocktail inhibitor (10 mg/ml aprotinin, 50 g/ml leupeptin, and 1.6 mmol/L phenylmethylsulfonyl fluoride). To prepare soluble *T. gondii* antigen (STAg), the parasite suspension was lysed by five sonication cycles (60 Hz for 1 min each) on ice. After centrifugation (10,000 g, 2 h, 4°C) supernatants were collected and sterilized by filtration through a 0.2 μm-pore size membrane (Corning Costar Corp., Cambridge, MA). The protein concentration was determined by Bradford (Quick Start™ Protein Assay, Bio-Rad laboratories, Hercules, CA) and aliquots were stored at −80°C until use.

### Patients and Study Strategy

From 1,083 pregnant women screened for toxoplasmosis, we recruited 11 of them who agreed to participate and met criteria for further analysis. They were patients of the “Instituto Nacional de Perinatología-Isidro Espinosa de los Reyes” (third level hospital) or the “Centro de Salud-Dr. Gustavo A. Rovirosa Pérez,” at Mexico City, Mexico. These volunteers ranged in age from 18 to 38 years and most of them (73%) were in the first half of pregnancy (Table 1). They had no diagnosis of other chronic or acute infection, or autoimmune disease according to the clinical profile records; none of them received any specific drug treatment at the sample collection time, but as soon as the diagnosis was confirmed, they were all treated up to delivery by standard schemes with spiramycin or pyrimethamine (1). All but one case (11, see Table 1) were screened by inhouse specific IgG-ELISA and confirmed by IgG and IgM western blot (WB). Then, the infection acute or subacute phase was supported by serological and molecular tests, i.e., seroconversion, increase of total IgG antibodies (abs) in serial samples, low IgG avidity test, and/or *B1* gene detection by qPCR; all these procedures had been standardized by us previously and described in detail elsewhere (11–14). To classify the mothers in non-transmitters or transmitters, we followed their gestations until delivery, and confirmed or discarded congenital infection by clinical and laboratory tests in their children, as previously reported (15). Briefly, the newborns were tested for IgM, IgA abs by ELISA; also, their WB IgG pattern was compared to that of their mothers in order to look for neo-antibodies; finally, qPCR was also performed. In one case (11, Table 1) diagnosis of congenital toxoplasmosis (CT) was confirmed by positive qPCR in the fetal blood taken through cordocentesis, due to suspicion of fetal infection at the hospital. All except one infected mother (case 11, Table 1) resulted positive to specific anti-*Toxoplasma* IgG in the first sample by ELISA, while 80% of the non-transmitter and 66% of the transmitter groups were positive to IgM.

**Table 1:** Comparison serological, clinical, and immunological profiles of between *T. gondii* transmitter and non-transmitter pregnant women.

**Case number**	**Age (years)**	**GWD**	**IgG avidity**	**WB IgM**	**WB IgG**	**Congenital transmission**	**Current pregnancy problems**	**History of obstetric problems**	**IgG1**	**IgG2**	**IgG3**	**IgG4**	**IgA**	**Lym**^**∞**^	**CD3**	**CD4**	**CD8**	**CD19**	**IFN-γ**	**TGF-β**
1^*^	36	7	Low	−	+	No	–	–	**+**	−	−	−	−	−	**+**	**+**	**+**	**+**	**+**	**+**
2	29	12	Low	−	+	No	–	Moebius	**+**	**+**	−	−	−	**+**	**+**	**+**	**+**	**+**	**+**	**+**
3	21	12	Gray zone	+	+	No	–	Miscarriage	**+**	−	**+**	−	−	**+**	**+**	**+**	**+**	**+**	−	**+**
4	33	14	Gray zone	+	+	No	–	Miscarriage	**+**	−	**+**	−	−							
5	30	16	Gray zone	+	+	No	–	–	**+**	−	−	−	−							
6	33	30	Gray zone	+	+	No	–	–	**+**	**+**	−	**+**	**+**	−	**+**	−	−	−	−	
Mean = >	28	15		4/6	6/6			Positive (%) = >	100	33	33	17	17	50	100	75	75	75	50	100
Median = >	30	13						Median = >	1.6	0.9	1.0	0.8	0.9	1.6	1.5	2.2	2.4	2.9	1.0	1.3
7	–	11	Low	+	+	Yes	Preeclampsia, hepatic cholestasis	–	**+**	**+**	**+**	−	**+**							
8	38	12	Low	+	+	Yes	–	Miscarriage and other baby with heart malformations	**+**	**+**	**+**	−	**+**	**+**	**+**	**+**	**+**	**+**	**+**	−
9	32	12	Low	−	+	Yes	–	Preeclampsia	**+**	**+**	**+**	**+**	**+**	**+**	**+**	**+**	**+**	**+**	**+**	**+**
10	–	24	Low	+	+	Yes	–	–	**+**	−	**+**	**+**	−	**+**	**+**	**+**	**+**	**+**	**+**	−
11	27	31	Sc^**^	−	−	Yes	Hydrops fetalis, polyhydramnios	–	**+**	**+**	**+**	**+**	**+**							
Mean = >	28	17		4/5	4/5^**^			Positive (%) = >	100	**80**^**+**^	**100**^**+**^	**60**^**+**^	**80**^**+**^	**100**^**+**^	100	**100**^**+**^	**100**^**+**^	**100**^**+**^	**100**^**+**^	**33**^**+**^
Median = >	32	13						Median = >	1.4	**1.3**^**$**^	**1.1**^**$**^	1.0	**1.1**^**$**^	**4.0**^**$**^	**2.8**^**$**^	6.4	2.7	4.4	1.0	0.8

*GWD, gestational week at diagnosis*.

∞ Total lymphocyte population;

* Case confirmed by positive PCR in maternal blood;

** *Sc: one mother seroconverted in the second sample; the fetus tissues were positive by PCR. Statistical analysis comparing Transmitters vs. Non-transmitters*:

+ Fisher's exact test p ≤ 0.0001 for comparison of frequencies;

$ *Mann-Whitney U-test, p ≤ 0.05, for medians. Bold values remark statistically different values in the transmitters compared to the non transmitters group*.

In the confirmatory sample, obtained no more than 15 days after the first one during pregnancy, we determined the levels of specific anti-*T. gondii* IgG1-4 subclasses and IgA abs by inhouse ELISA, in addition to Peripheral Blood Mononuclear Cells (PBMCs) *in vitro* proliferation and cytokine production after specific stimulation when a sample was enough.

### Anti-*T. gondii* Antibody Detection and Avidity Test

Anti-*T. gondii* total IgG/avidity test and class/subclass antibodies detection in serum were performed using previously validated indirect homemade ELISAs (13, 16). Briefly, STAg was coated overnight at 4°C in high binding plates (Maxisorp, Nunc Co, Rochester, NY) at 1 μg/mL in 0.1 M carbonate buffer. Blocked with 1% bovine serum albumin in PBS-tween 20 and incubated with serum in the presence or not of 6 M urea. Development was done with a commercial HRP-anti human IgG diluted 1:1000 in PBS-tween 20 and the substrate/chromogen solution with O-phenylendiamine, until color development. Avidity percentage was calculated dividing the absorbance of the wells, treated with urea and untreated wells; cutoffs for avidity were as follows: below 51% acute, 51–65% gray zone, and above 65% chronic (13). For IgG class/subclass, plates were coated overnight with 5 μg/mL of the STAg, blocked with 1% BSA -PBS, sera diluted 1:500 for IgG, 1:250 for IgG1, and 1:125 for IgG2-4, and IgA in PBS-0.05% Tween 20. Developed with peroxidase-labeled goat anti-human IgG or IgA, or biotinylated monoclonal abs against each IgG subclass, at optimal dilutions (16). For IgG subclasses, HRP-Streptavidin was used as final step, before the substrate/chromogen solution with O-phenylendiamine was added; 0.1 N sulfuric acid was used to stop enzymatic activity in all cases (16). Finally, plates were read at 492 nm. The results are expressed as the Reactivity Index (RI), that was calculated dividing the mean absorbance of duplicates of each sample by the cut-off value for each immunoglobulin (mean plus three standard deviations of six low, medium, and high negative controls). An RI ≥1.0 was considered positive (13).

### Peripheral Blood Samples, Cell Culture and Proliferation Testing

Approximately 5 mL of blood from participants, was obtained into EDTA-Vacutainer tubes. To obtain PBMCs, peripheral blood was subjected to Ficoll-Hypaque separation (GE Healthcare, Piscataway, NJ, USA). Then, PBMCs were labeled with 0.2 μM CFSE (Molecular Probes/Invitrogen Detection Technologies, Eugene, OR) for 5 min, the reaction was stopped by adding cold RPMI supplemented with 10% fetal calf serum (FCS) (Gibco, Carlsband, CA, USA). Cells were washed exhaustively with cold RPMI supplemented with 10% FCS, seeded into 96-well plates at a concentration of 1 × 10^6^ cells/mL and stimulated with 10 μg/mL of the STAg in triplicate, and then incubated at 37°C in 5% CO_2_/95% air atmosphere, for 72 h for cytokine detection, and 120 h for cell proliferation assays. Concanavalin-A [5 μg/mL] and medium alone were used as controls, positive, and negative, respectively. After the stimulation period, CFSE-labeled PBMCs were collected and stained with the following antibodies: CD3/APC-Cy7 (SK7), CD4/APC(OKT4), CD8/PERCP-Cy5 (RPA-T8), CD19/PE-Cy7 (HIB19) (BioLegend, San Diego, CA, USA). All samples were analyzed in a FACS-Aria flow cytometer (BD Biosciences, San Jose, CA, USA) using FlowJo (version 7) software (Tree Star Inc., Ashland, OR, USA). For the analysis, 1 × 10^4^ events were collected. Relative proliferation index was calculated by dividing proliferation percentage of STAg-stimulated PBMCs by the proliferation percentage of non-stimulated PBMCs.

### Cytokine Detection in PBMCs Culture Supernatants

Cytokine concentration in the supernatants obtained from PBMCs cultures, was quantified using the human inflammatory CBA kit, human Th1/Th2 CBA kit, and the human TGF-β1 Single Plex Flex set (BD Biosciences, San Jose, CA). The CBA analysis was performed with the FCAP Array Software v3.0 (BD Biosciences), from data obtained in a BD FACSAria flow cytometer (BD Biosciences). Production index for each cytokine tested was calculated dividing cytokine concentration from STAg-stimulated PBMCs by the cytokine concentration of the non-stimulated PBMCs.

### Data Analysis and Statistics

Antibody reactivity, cytokine production, and proliferation indexes were compared between non-transmitter and transmitter groups, determining the statistical significance by comparison of ranks using the Mann-Whitney *U*-test and comparing proportions between groups using the Fisher's exact test. A *P* < 0.05 was considered significant. The GraphPad Prism version 7.0d for Macintosh, GraphPad Software, La Jolla California USA, www.graphpad.com, was used for these purposes.

## Results

As it can be seen in Table 1, those pregnant women who congenitally transmitted the parasite to their newborns generally presented a stronger and more variable humoral and cellular immune response than their non-transmitter counterparts. The exceptions were IgG1 abs and proliferation of CD3^+^ lymphocytes, for which no difference was found between groups. IgG2,3,4 and IgA abs were present in a greater proportion of transmitters. Likewise, the proliferation of total lymphocytes, as well as of the CD4^+^, CD8^+^, and CD19^+^ subpopulations, was proportionally higher in the transmitter group (Table 1). Although we measured a broad range of cytokines such as IL-1β, IL-2, IL-4, IL-6, IL-8, IL-10, IL-12, TNF-α, IFN-γ, and TGF-β in culture supernatants from PBMCs stimulated with the STAg, we found a higher number of positive cases to TGF-β in non-transmitter than in transmitter women we could test.

## Discussion

In this work, we measured IgG1-4, and IgA abs in serum, as well as *in vitro* PBMCs proliferation and cytokine production in response to STAg in infected women during pregnancy, and this response was analyzed as a function of parasite transmission to their newborns.

Herein, we found similar results of a previous work in which we did not encounter different proportions of positive mothers to IgG1 abs between groups (11). But, we found that IgG2-3 subclasses and IgA during pregnancy were more frequent and some of them at higher levels in transmitters than non-transmitters. Detection of any subclass before the end of pregnancy may reflect an anti-parasite immune response closer to the point of infection than those found at birth, especially for women with acute/subacute response (17–19). Interestingly, the four IgG subclasses are transported by the FcRn throughout the placenta since the first trimester (20, 21) and as it has been demonstrated by our group, each woman can develop a specific response in which one or two IgG subclasses predominate (16). Our data challenge the idea that maternal anti-*T. gondii* IgG abs cross the placenta and protect the offspring from infections *in utero* and during early life (22). Moreover, FcRn is expressed by human microvasculature endothelial cells, facilitating a phenomenon that may be present in acquired toxoplasmosis too, facilitating body dispersion of the parasite (23). In general, our results showed that a more variable antibody response, might be directly or indirectly related to transmission of the parasite; if this is true, the immune response mediated by the maternal antibodies might be fetus-averse, not friend; at least, they seem non-protective.

Additionally, initiation and sustained strong T-cell-mediated immunity is critical in preventing proliferation and dissemination of the parasite in acquired toxoplasmosis (4, 5). There is limited information about the maternal cell-mediated immunity during pregnancy against *T. gondii* in humans, and it has not been related to transmission (6–9). In this work, we found an increased proportion of specific anti STAg proliferation of total CD4^+^, CD8^+^, and CD19^+^ lymphocytes in the group of transmitter mothers. Holfheld et al. showed that the percentage of total T cells in mothers infected with the parasite is increased in peripheral blood, especially CD3 and CD8 positive cells, and consistent with our work, they suggested that mothers whose fetuses are infected, seem to have more important alterations in the percentages of the different subsets when compared to women whose fetuses are not infected; however, they searched for total, i.e., non-*T. gondii* specific, CD3^+^ and CD8^+^ cells (6). Another report by Fatoohi et al. showed that in women infected during pregnancy, CD4^+^ cells are the main responders against the STAg (7). More recently, it has been demonstrated that in *T. gondii* primo-infected pregnant women, there is an increased proliferation of PBMCs, principally CD3^+^CD4^+^ (8) and showed an important role of γδ T lymphocytes producing IL-17 (9); nevertheless, in these studies the potential impact of mother's immune response on congenital transmission was not determined, but they focused in comparing the cellular immune response in parturient vs. non-pregnant women (8, 9).

Others have reported that *T. gondii* infected pregnant woman can produce IFN-γ (5, 7–9, 24), but in this work we found transmitters as positive producers of this cytokine and additionally, lower producers of TGF-β. As it is well-known, this parasite induces high levels of IFN-γ during infection as a result of early T-cell stimulation (25). IFN-γ production promotes a robust inflammatory environment that limits infection in the mother‘s tissues (26). However, during pregnancy, there is evidence suggesting a paradoxical role of Th1 pro-inflammatory molecules, associating them to vertical transmission, or induction of damage to the offspring in murine models: IFN-γ inhibition during pregnancy has been associated with a lower proportion of infected fetuses in mice (24, 27, 28). This was explained by the “Trojan horse” hypothesis, in which parasite dissemination depends on the ability to infect highly motile dendritic cells and monocytes/macrophages recruited by the pro-inflammatory environment to the site of infection, and spread into several tissues, including the brain, even if the parasite infection is acquired orally (29, 30). It has been demonstrated that STAg can induce the expression of MIF in an IFN-γ-dependent manner, and furthermore, MIF is able to induce ICAM-1 expression (31, 32). Interestingly, ICAM-1 mediates cellular adhesion of *T. gondii* infected monocytes to human trophoblasts *in vitro* (32), and has also been described as MIC2 ligand, a *T. gondii* adhesin (33). In general, the Th1 environment orchestrated by IFN-γ, can attract infected cells or even induce the expression of pro-inflammatory proteins that may facilitate maternal-fetal interface parasite direct adhesion, probably facilitating its infection, and passage throughout the placenta (31). Together with an overproduction of Th1 pro-inflammatory molecules, a poorly regulated environment is detrimental for pregnancy. TGF-β is an important Th1 antagonist cytokine, which plays an important role in the maintenance of pregnancy. Interestingly, *T. gondii* infection inhibits TGF-β production at the fetal-maternal interface in mice, provoking adverse outcome (34). Moreover, TGF-β can modulate mothers' immune response during pregnancy, by increasing Treg differentiation (35) and it has been reported that *T. gondii* infection during pregnancy in mice reduces splenic and placental CD4^+^CD25^+^ regulatory T cells (36). More recently, Zhao et al. showed that *T. gondii-*infected pregnant mice treated with TGF-β improved adverse pregnancy outcomes, while TGF-β neutralization produced serious hemorrhage, more dilated uterine spiral arteries, and decreased local Treg cell numbers (37); these data are in agreement with our results and may suggest that an increased inflammation induced by *T. gondii* could promote congenital transmission in a poorly regulated environment due to low TGF-β production, favoring systemically abs production and lymphocytes proliferation that may be involved in dissemination and transmission of the parasite.

Only one mother of the non-transmitter group had circulating parasite DNA, as demonstrated by qPCR, so the parasite burden in blood did not seem to account for higher transmission risk. Since we recruited mothers who had abs of low/gray zone avidity, our results could be reflecting the acute response that accompanies parasite proliferation and placental crossing, but even in this case, it can be stated that the response which is protective for the adult host, is ineffective to prevent vertical transmission. Our results only demonstrate a potential relation between maternal immune profile during pregnancy and *T. gondii* vertical transmission, but since they are congruent with results in animal models and previous indirect results in humans mentioned above, we think that a rather strong and non-regulated maternal immune response during pregnancy is directly or indirectly related to *T. gondii* congenital transmission, which deserves more investigation.

### Limitations of the Study

The main limitation of this study was the small number of cases, which hindered our ability to have stronger statistical support. Studies of Treg cells and cytokines not included in the present study could add important information about this complex form of toxoplasmosis.

## Author Contributions

DC and RF-D conceived experimental design and wrote the project to which these data belong. JM-G recruited part of the pregnant women studied. FG-C, YF-G, IC-S, LO-A, and HL-P conducted the laboratory experiments. FG-C and DC reviewed and analyzed the data. FG-C wrote the paper. IC-S, LO-A, RF-D, JM-G, and DC revised it. All authors read the final version of the manuscript and approved it for publication.

### Conflict of Interest Statement

The authors declare that the research was conducted in the absence of any commercial or financial relationships that could be construed as a potential conflict of interest.
